# Oligomerization of Indole Derivatives with Incorporation of Thiols

**DOI:** 10.3390/molecules13081846

**Published:** 2008-08-26

**Authors:** Felikss Mutulis, Adolf Gogoll, Ilze Mutule, Sviatlana Yahorava, Aleh Yahorau, Edvards Liepinsh, Jarl E.S. Wikberg

**Affiliations:** 1 Department of Pharmaceutical Biosciences, Division of Pharmaceutical Pharmacology, Uppsala Biomedical center, Uppsala University, S-751 24, Uppsala, Sweden; E-mails: Sviatlana.Yahorava@farmbio.uu.se; Aleh.Yahorau@farmbio.uu.se; Jarl.Wikberg@farmbio.uu.se; 2 Latvian Institute of Organic Synthesis, Aizkraukles 21, LV-1006, Riga, Latvia; E-mail: ilzem@osi.lv; 3 Department of Biochemistry and Organic Chemistry, Box 576, Uppsala University, S-751 23, Uppsala, Sweden; E-mail: Adolf.Gogoll@biorg.uu.se

**Keywords:** Side reaction, oligomerization of indole derivatives, incorporation of thiols, regioselectivity, NMR study

## Abstract

Two molecules of indole derivative, e.g. indole-5-carboxylic acid, reacted with one molecule of thiol, e.g. 1,2-ethanedithiol, in the presence of trifluoroacetic acid to yield adducts such as 3-[2-(2-amino-5-carboxyphenyl)-1-(2-mercaptoethylthio)ethyl]-1*H*-indole-5-carboxylic acid. Parallel formation of dimers, such as 2,3-dihydro-1*H*,1'*H*-2,3'-biindole-5,5'-dicarboxylic acid and trimers, such as 3,3'-[2-(2-amino-5-carboxy-phenyl)ethane-1,1-diyl]bis(1*H*-indole-5-carboxylic acid) of the indole derivatives was also observed. Reaction of a mixture of indole and indole-5-carboxylic acid with 2-phenylethanethiol proceeded in a regioselective way, affording 3-[2-(2-aminophenyl)-1-(phenethylthio)ethyl]-1*H*-indole-5-carboxylic acid. An additional product of this reaction was 3-[2-(2-aminophenyl)-1-(phenethylthio)ethyl]-2,3-dihydro-1*H*,1'*H*-2,3'-biindole-5'-carboxylic acid, which upon standing in DMSO-d_6_ solution gave 3-[2-(2-aminophenyl)-1-(phenethylthio)ethyl]-1*H*,1'*H*-2,3'-biindole-5'-carboxylic acid. Structures of all compounds were elucidated by NMR, and a mechanism for their formation was suggested.

## Introduction

Multistage solid phase organic synthesis, which excludes isolation of intermediates steps, is an attractive method for the preparation of diverse chemical substances [1]. However, a high yield of desired product at each synthetic stage is particularly important for these methods [2], as various side reactions, lowering the chemical yield of the individual synthetic steps, are the main limitation of the method [3]. In order to make the method more efficient, a deeper understanding of these unwanted processes is desirable.

**Scheme 1:**
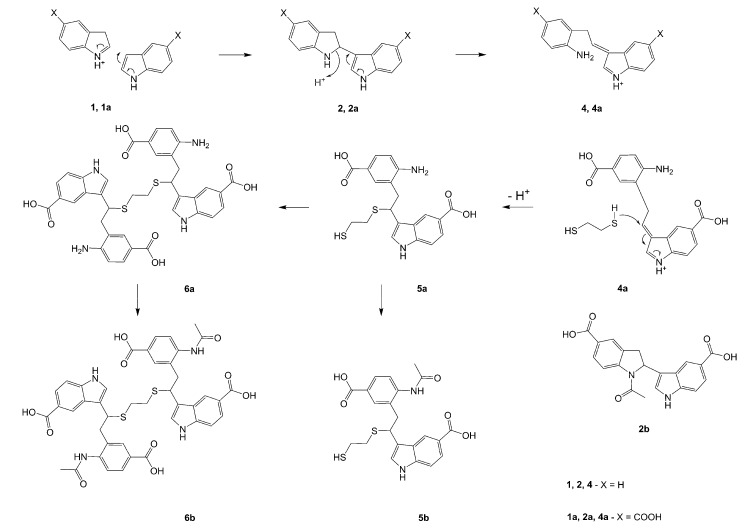
Incorporation of thiols. Proposed mechanism and related substances.

In our previous investigation of indole-5-carboxylic acid derivatives attached to carboxylated Wang polymer [4], LC/MS analysis after the cleavage step using trifluoroacetic acid and 1,2-ethanedithiol mixture unexpectedly showed the presence of compounds whose molecular mass corresponded to dimerization of the expected products with addition of 1,2-ethanedithiol.

**Figure 1:**
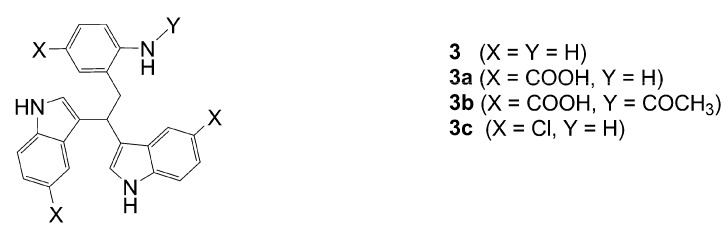
Indole trimers.

Dimers [5,6,7,8,9,10], trimers [8, 11,12,13,14,15,16,17] and tetramers [7, 14, 15, 17] of indole and its derivatives are described in a number of research articles and patents. It is well documented that indole (**1**) forms the indole dimer 2,3-dihydro-1*H*,1'*H*-2,3'-biindole [6, 15] (**2**, Scheme 1) and trimer 2-[2,2-di(1*H*-indol-3-yl)ethyl]-aniline [5, 12, 15] (**3**, Figure 1) under various acidic conditions.

## Results and Discussion

To investigate the aforementioned unknown side reaction, in the present study we tried to prepare a similar compound from indole-5-carboxylic acid itself. Indole-5-carboxylic acid (**1a**, Scheme 1) was treated with trifluoroacetic acid and 1,2-ethanedithiol mixture. Indeed, the expected dimerization and addition reactions furnished the desired product. In addition, according to LC/MS, along with the dimer and trimer of indole-5-carboxylic acid, a product, whose molecular mass corresponded to the sum of mass of four molecules of indole-5-carboxylic acid and one molecule 1,2-ethanedithiol was detected.

A detailed NMR investigation of the products was performed using ^1^H [18], COSY [18, 19], proton decoupled ^13^C [18, 20], HETRES [21] and selective long range INEPT [22] experiments, and evaluation of the resulting NMR data confirmed proposed structures **2a **(Scheme 1), **3a **(Figure 1), **5a **and **6a **(Scheme 1). Moreover, as additional proof of the correctness of the proposed structures, their corresponding acetylated derivatives **2b** (Scheme 1), **3b **(Figure 1), **5b** and **6b** (Scheme 1) were also prepared and their structures investigated.

1,2-Ethanedithiol is known to be a strong nucleophilic reagent, widely used as the most effective scavenger of carbonium ions [23]. Incorporation of dithiol could be explained in terms of the mechanism proposed by Smith [11] and Sundberg [24] for oligomerization of indole (Scheme 1). We suggest that ethanedithiol reacts with the indole-5-carboxylic acid dimer **4a** (Scheme 1) forming an ethanedithiol adduct **5a**. Competitive attachment of the third molecule of indole-5-carboxylic acid leads to parallel formation of trimer **3a **(Figure 1). It is logical to assume that in a way similar to formation of **5a**, the SH group in **5a** reacts further with the protonated dimer **4a** yielding a symmetric structure **6a **(Scheme 1).

**Scheme 2:**
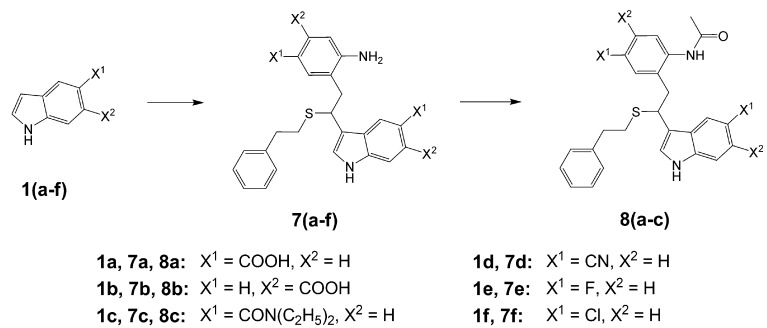
Incorporation of 2-phenylethanethiol.

Further, we investigated the influence of substitution pattern in the indole on the course of the oligomerization. Replacing 1,2-ethanedithiol with 2-phenylethanethiol and varying substituents in the indole system we obtained products **7a-f** analogous to **5a** (Scheme 2). Indole-5-carboxylic acid, its diethylamide [24], indole-6-carboxylic acid, 5-cyanoindole, 5-fluoroindole and 5-chloroindole were used. Acetylated derivatives of 2-phenylethanethiol adducts **8a-c** (Scheme 2) were prepared to assist in the spectroscopic structure assignments. Incorporation of thiols was accompanied by formation of ample quantities of dimers of indoles like **2a**, acetylated derivative **2b** (Scheme 1) and indole trimers like **3a**, acetylated derivative **3b**, and **3c** (Figure 1) (according to LC/MS data).

**Scheme 3:**
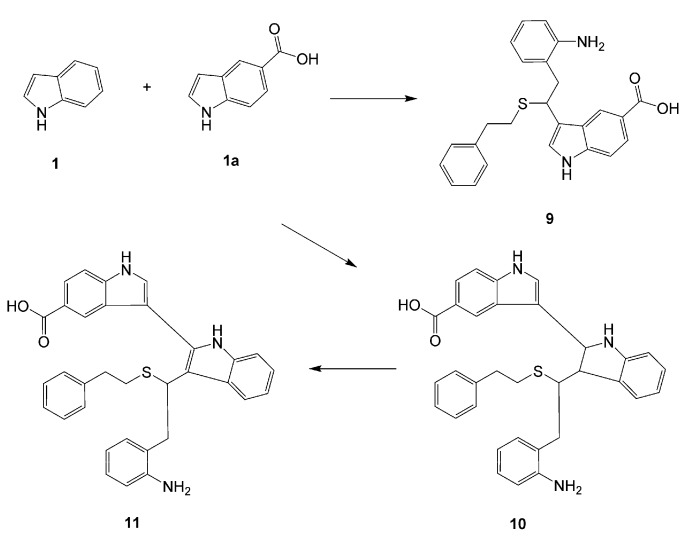
Introduction of unsubstituted indole.

It was not possible to obtain an adduct from unsubstituted indole and 1,2-ethanedithiol under typical reaction conditions. Incorporation of this indole was only possible when it was used in a mixture with indole-5-carboxylic acid. In addition, it was noted that the reaction proceeded in a regioselective way yielding **9** (Scheme 3). This reactivity pattern of unsubstituted indole could be explained on the basis of mechanism shown in Scheme 1. Michael additions of thiols are known to be facilitated by the electrodeficiency of the participating alkenes [26]. Electron acceptors attached to the benzene ring of indole should decrease the electron density on the carbon atom being attacked by thiols in structures like **4a** (Scheme 1), thereby promoting the reaction.

An additional indole oligomerization product **10**, corresponding to the combination of two molecules of indole, one molecule of indole-5-carboxylic acid and one molecule of 2-phenylethane-thiol was isolated from the reaction mixture (Scheme 3). Upon standing in DMSO-d_6_ solution for one week, the indoline structure in **10** was oxidized quantitatively to yield indole **11**.

Yields for the thiol incorporation products reached 30% (Table 1). They were higher if carbonyl (compounds **5a**, **7a-c**, **9**) or nitrile (**7d**) groups were attached to the starting indole molecule. Much less reactive were halogenated indoles (products **7e** and **7f**). Particular low were the yields for tetramer **6a** and trimer **10**.

**Table 1:** Representative yields for products of incorporation of thiols.

Compound	5a 6a 7a 7b 7c 7d 7e 7f 9 10
**Yield, %**	33 1.3 19 32 30 15 2 5 19 1

## Conclusions

In summary, we have discovered a previously unknown indole oligomerization with incorporation of thiols, which has potential synthetic utility. The reaction proceeds with parallel formation of indole 3,3´-trimers; both processes require indole derivatives that are free of substitution at both the 2- and 3- positions. The inclusion of electronegative substituents in benzene ring of indole is another crucial factor that makes incorporation of thiols possible. Adducts similar to those reported herein might be of interest as, e.g., potential enzyme inhibitors.

## Experimental

### General

Reagents were obtained from Aldrich or Fluka. Evaporations of solvents were carried out on a vacuum rotary evaporator at 30 ^o^C and 20 mbar. TLC was performed using Merck Silica gel 60 F 254 glass plates; flash chromatography was performed using Merck Silica gel (70-230 mesh, pore size 60Å). LC/MS was performed on a Perkin Elmer PE SCIEX API 150EX instrument with a Turboionspray Ion Source and equipped with a Dr. Maisch Reprosil-Pur C18-AQ, 5 μ, 150 × 3 mm HPLC column, using a water and acetonitrile gradient with 5 mM ammonium acetate additive. Semi-preparative HPLC was carried out on a LKB system consisting of a 2150 HPLC Pump, 2152 LC Controller and 2151 Variable Wavelength Monitor and Vydac RP C_18_ column (10 × 250 mm, 90 Å, 201HS1010), the eluent being an appropriate concentration of MeCN in water + 0.1% TFA, flow rate 5 mL/min, detection at 280 nm. Freeze-drying was performed at 0.1 milibar on a Lyovac GT2 Freeze-Dryer (Finn-Aqua) equipped with a Busch 010-112 vacuum pump and a liquid nitrogen trap. Exact molecular masses were determined on a Micromass Q-Tof2 mass spectrometer equipped with an electrospray ion source. ^1^H-NMR spectra were recorded on a Jeol JNM-EX270 or Jeol JNM-EX400, Bruker DMX-600 spectrometer equipped with a cryoprobe or a Bruker DMX-500 spectrometer. Chemical shifts are reported in ppm relative to residual solvent signal [δ (^1^H) 2.50 ppm, δ (^13^C) 39.5 ppm]. Two-dimensional spectra recorded included COLOC, HETRES, selective long range INEPT, COSY, ROESY, sensitivity-enhanced ^13^C-HSQC and ^13^C-^1^H HMBC. ROESY mixing time was 0.1 s. Pulsed-field gradients were used for all ^13^C correlation spectra. ^13^C-HMBC spectra were recorded with coupling evolution delay for the generation of multiple-bond correlations set to 62.5 ms. ROESY, ^13^C-HSQC and ^13^C-^1^H HMBC spectra were run with 4096*1024 points data matrix, giving τ_2max_ = 250 ms for ^1^H nucleus in acquisition dimension and τ_1max_ = 200 ms for ^1^H or τ_1max_ = 50 ms for ^13^C for indirect dimension; prior to Fourier transform the data matrix was zero-filled twice and multiplication by shifted sine-bell window function applied. For ^1^H-^13^C HMBC the magnitude spectra were calculated.

### Procedure A: Preparation of compounds **2a**, **3a**, **5a** and **6a**

Indole-5-carboxylic acid (**1a**, 370 mg, 2.3 mmol) was dissolved in a mixture of trifluoroacetic acid (5 mL) and 1,2-ethanedithiol (1 mL). After 1 h at room temperature the mixture was evaporated without heating under water aspirator pump vacuum. Dry ether-hexane (1:1, 50 mL) was added to the residue and the white precipitate formed was filtered off, washed with 1:1 dry ether-hexane and dried *in vacuo*. The raw product was next dissolved in 24 % MeCN in water, centrifuged and the clear solution applied in several portions onto an HPLC semipreparative column (10 x 250 mm, Vydac RP C_18_ 90Å Pharmaceutical 201HS1010), eluent - 20 % MeCN in water + 0.1% TFA, flow 5 mL/min, detection at 280 nm. Eluate fractions, containing putative **2a **(RT 5 min), **3a** (RT 8 min) and **5a** (RT 17 min) were separately pooled. Next the eluent was changed to 80 % MeCN in water + 0.1% TFA and a peak containing **6a** was collected. Freeze drying provided pure **2a **(15 mg, 4 %), **5a** (156 mg, 33 %) and partially purified **3a** and **6a** as powders. The fraction containing **3a** was dissolved in 16 % MeCN in water and applied in several portions onto an HPLC column (4 × 250 mm, Merck Hibar Lichrosorb RP18, 10μm), eluent - 16 % MeCN in water + 0.1% TFA, flow 2 mL/min, detection at 220 nm. Eluate fractions containing pure **3a** were pooled and lyophilized to give a yellow powder. (yield 15 mg, 4 %). The fraction containing **6a** was purified again on the Vydac column (eluent - 22 % MeCN in water + 0.1% TFA), followed by purification on the Merck column (eluent - 22 % MeCN in water + 0.1% TFA) to give, after freeze drying, a white powder (2.1 mg, 0.3 %).

### Characterization data

*2,3-Dihydro-2,3'-biindole-5,5'-dicarboxylic acid* (**2a**): HRMS: M+H^+^ (C_18_H_15_N_2_O_4_) 323.1019, calculated 323.1032; M-H^-^ (C_18_H_13_N_2_O_4_) 321.0861, calculated 321.0876; Elemental analysis: found, %: N 8.2, C 64.9, H 4.8; calculated for 3C_18_H_14_N_2_O_4_·2H_2_O, %: N 8.38, C 64.67, H 4.62.


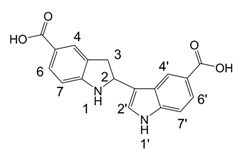


^1^H-NMR (400 MHz, DMSO-d_6_, 25°C): δ 3.03 (dd, *J* = 16.0, 8.8 Hz, 1H, H-3), 3.43 (dd, *J* = 16.0, 9.2 Hz, 1H, H-3), 5.28 (t, *J* = 9.2 Hz, 1H, H-2), 6.50 (d, *J* = 8.0 Hz, 1H, H-7), 7.36 (d, *J* = 2.0 Hz, 1H, H-4), 7.53 (d, *J* = 8.4 Hz, 1H), 7.58 (m, 1H), 7.61 (dd, *J* = 8.0, 1.6 Hz, 1H, H-6), 7.81 (dd, *J* = 8.4, 1.6 Hz, 1H), 7.96 (m, 1H), 11.26 (d, *J* = 1.6 Hz, 1H, H-1), 11.79 (d, *J* = 2.0 Hz, 1H, H-1’), 12.3 (m, 2H, 2COOH)

*3,3'-[2-(2-Amino-5-carboxyphenyl)ethane-1,1-diyl]bis(1H-indole-5-carboxylic acid* (**3a**): HRMS: M+H^+^ (C_27_H_22_N_3_O_6_) 484.1504, calculated 484.1508; M-H^-^ (C_27_H_20_N_3_O_6_) 482.1331, calculated 482.1352; Elemental analysis: found, %: N 7.7, C 58.5, H 5.4; calculated for C_27_H_21_N_3_O_6_·4H_2_O, %: N 7.56, C 58.37, H 5.26.


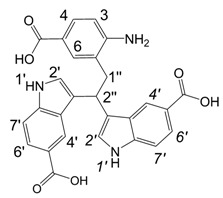


^1^H-NMR (270 MHz, DMSO-d_6_, 25°C): δ 3.22 (m, 2H, 2H-1’’), 4.93 (m, 1H, H-2’’), 6.58 (d, *J* = 8.6 Hz, 1H, H-3), 7.31 (d, *J* = 8.6 Hz, 2H, H-7’, H-*7’*), 7.33, 7.34 (2m, 2H, H-2’, H-*2’*), 7.35 (m, 1H, H-4), 7.40 (m, 1H, H-6), 7.59 (dd, *J* = 8.6 Hz, 1.6 Hz, 2H, H-6’, H-*6’*), 8.08 (d, *J* = 1.7 Hz, 2H, H-4’, H-*4’*), 11.14 (d, *J* = 1.9 Hz, 2H, H-1’, H-*1’*).

*3-(2-(2-Amino-5-carboxyphenyl)-1-(2-mercaptoethylthio)ethyl)-1H-indole)-5-carboxylic acid* (**5a**): HRMS: M+H^+^ (C_20_H_21_N_2_O_4_S_2_) 417.0957, calculated 417.0943; M-H^-^ (C_20_H_19_N_2_O_4_S_2_) 415.0794, calculated 415.0787; Elemental analysis: found, %: N 6.5, C 57.8, H 5.0; calculated for C_20_H_20_N_2_O_4_S_2_, %: N 6.73, C 57.67, H 4.84.


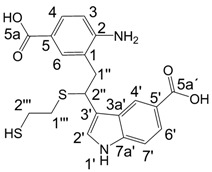


^1^H-NMR (400 MHz, DMSO-d_6_, 25°C): δ 2.34(m, 1H, SH), 2.5 (m, 4H, 2H-1’’’, 2H-2’’’), 3.22 (m, 2H, 2H-1’’), 4.67(m, 1H, H-2’’), 6.61 (d, *J* = 8.3 Hz, 1H, H-3), 7.39 (d, *J* = 8.6 Hz, 1H, H-7’), 7.45 (d, *J* = 2.2 Hz, 1H, H-2’), 7.46 (dd, *J* = 8.3 Hz, 2.0 Hz, 1H, H-4), 7.53 (d, *J* = 2.0 Hz, 1H, H-6), 7.71 (dd, *J* = 8.6 Hz, 1.6 Hz, 1H, H-6’), 8.43 (d, *J* = 1.6 Hz, 1H, H-4’), 11.27 (d, *J* = 2.2 Hz, 1H, H-1’); ^13^C-NMR (100 MHz, DMSO-d_6_, 25°C): δ 24.8 (C-2’’’), 35.0 (C-1’’’), 37.2 (C-1’’), 40.0 (C-2’’), 112.0 (C-7’), 116.6 (C-3’), 119.0 (C-5), 121.5 (C-5’), 122.8 (C-4’), 123.1 (C-1), 123.1 (C-6’), 125.9 (C-3a’), 126.0 (C-2’), 129.6 (C-4), 132.8 (C-6), 139.6 (C-7a’), 150.0 (C-2), 168.1 (C-5a), 169.1 (C-5a’).

*3,3'-{1,1'-[Ethane-1,2-diylbis(sulfane-diyl)]bis[2-(2-amino-5-carboxyphenyl)ethane-1,1-diyl]}bis(1H-indole-5-carboxylic acid)* (**6a**): HRMS: M+H^+ ^(C_38_H_35_N_4_O_8_S_2_) 739.1880, calculated 739.1896; M-H^-^ (C_38_H_33_N_4_O_8_S_2_) 737.1733, calculated 737.1740; Elemental analysis: found, %: N 5.3, C 47.9, H 4.2; calculated for C_38_H_34_N_4_O_8_S_2_·2CF_3_COOH·5H_2_O, %: N 5.30, C 47.73, H 4.39.


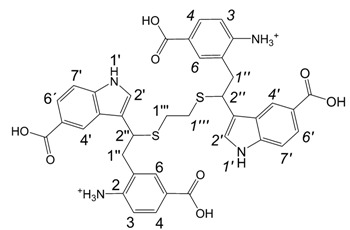


^1^H-NMR (270 MHz, DMSO-d6, 25°C): δ 2.3 (m, 4H, 2H-1’’’, 2H-*1’’’*), 3.13 (m, 4H, 2H-1’’, 2H-*1’’*), 4.55 (m, 2H, H-2’’, H-*2’’*), 6.55 (d, *J* = 8.6 Hz, 2H, H-3, H-*3*), 7.30, 7.31 (2d, *J* = 2.3 Hz, 2H, H-2’, H-*2’*), 7.35 (d, *J* = 8.6 Hz, 2H, H-7’, H-*7’*), 7.42-7.64 (m, 2H, H-4, H-*4*), 7.43-7.66 (m, 2H, H-6, H-*6*), 7.68 (dd, *J* = 8.6 Hz, 1.6 Hz, 2H, H-6’, H-*6’*), 8.38, 8.39 (2d, *J* = 1.6 Hz, 2H, H-4’, H-*4’*), 11.18 (d, *J* = 2.3 Hz, 2H, H-1’, H-*1’*).

*Procedure B:* Compounds **2a**, **3a**, **5a** and **6a** were also obtained by a modification of Procedure A, whereby the crude product was dissolved in chloroform and applied onto a glass column filled with silica gel (pore size 60Å, 70-230 mesh). The column was eluted with chloroform-methanol mixture, gradually changing its proportions from 20:1 to 1:4, and thereafter the elution was made with pure methanol. Eluate fractions containing pure **3a** and **5a** were separately pooled and evaporated. White crystalline products were obtained. Isolated yield of **3a** was 7%, and that of **5a** was 25%. (however, determination of the reaction products by HPLC provided the following yields: **2a**: 22%, **3a**: 27%, **5a**: 28% and **6a**: 1.3%).

**3a**: Elemental analysis. Found, %: N 7.6, C 64.7, H 5.3. Calculated for 3C_27_H_21_N_3_O_4_·4MeOH, %: N 7.99. C 64.67, H 5.04.

*2-(2,2-bis(5-Chloro-1H-indol-3-yl)ethyl)-4-chloroaniline* (**3c**). Compound **3c** was obtained from 5-chloroindole using Procedure A. Yield 63 %. HRMS: M+H^+^ (C_24_H_19_Cl_3_N_3_) 454.0637, calculated 454.0644; Elemental analysis: found, %: N 7.17, C 54.81, H 3.45; calculated for C_24_H_18_Cl_3_N_3_·CF_3_COOH, %: N 7.39, C 54.90, H 3.37.


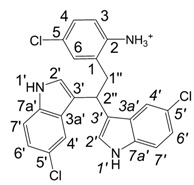


^1^H-NMR (400 MHz, DMSO-d_6_, 25°C): δ 3.36 (d, *J* = 7.9 Hz, 2H, 2H-1’’), 4.89 (t, *J* = 7.9 Hz, 1H, H-2’’), 6.78 (m, 1H, H-3), 6.93 (m, 2H, H-4, H-6), 6.99 (dd, *J* = 8.6 Hz, 2.2 Hz, 2H, H-6’, H-*6’*), 7.29 (d, *J* = 8.6 Hz, 2H, H-7’, H-*7’*), 7.42 (d, 2H, H-2’, H-*2’*), 7.49 (d, *J* = 2.2 Hz, 2H, H-4’, H-*4’*), 10.99 (d, *J* = 2.4 Hz, 2H, H-1’, H-*1’*); ^13^C-NMR (67.5 MHz, DMSO-d_6_, 25°C): δ 32.0 (C-2’’), 35.7 (C-1’’), 113.4 (C-7’, *C-7’*), 118.2 (C-3’, C-*3’*), 118.8 (C-4’, C-*4’*), 120.1 (C-3), 121.2 (C-6’, C-*6’*), 123.3 (C-5’, C-*5’*), 124.9 (C-1, C-2’, C-*2’*), 126.8 (C-6), 128.2 (C-3a’, C-*3a’*), 129.8 (C-4), 130.8 (C-5), 135.4 (C-7a’, C-*7a’*), 140.0 (C-2).

*3-[2-(2-Amino-5-carboxyphenyl)-1-(phenethylthio)ethyl]-1H-indole-5-carboxylic acid* (**7a**). This compound was prepared according to Procedure A using indole-5-carboxylic acid and 2-phenylethanethiol as starting materials. Yield 19%; HRMS: M+H^+^ (C_26_H_25_N_2_O_4_S) 461.1532, calculated 461.1535; Elemental analysis: found, %: N 5.82, C 65.44, H 5.31; calculated for C_26_H_24_N_2_O_4_S·H_2_O, %: N 5.85, C 65.25, H 5.48.


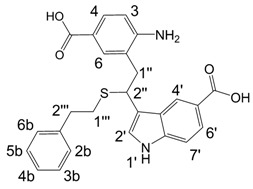


^1^H-NMR (270 MHz, DMSO-d_6_, 25°C): δ 2.39 and 2.58 (2m, 4H, 2H-1’’’, 2H-2’’’), 3.13-3.32 (m, 2H, 2H-1’’), 4.66 (t, *J* = 7.6 Hz, 1H, H-2’’), 6.59 (d, *J* = 8.5 Hz, 1H, H-3), 7.02 (XX´ part of AA´XX´system, 2H, H-2b, H-6b), 7.08-7.22 (m, 1H, H-4b), 7.08-7.22 (AA´ part of AA´XX´system, 2H, H-3b, H-5b), 7.37 (d, *J* = 8.6 Hz, 1H, H-7’), 7.45 (m, 2H, H-4, H-6), 7.53 (d, *J* = 2.3 Hz, 1H, H-2’), 7.69 (dd, *J* = 8.6 Hz, 1.6 Hz, 1H, H-6’), 8.43 (d, *J* = 1.6 Hz, 1H, H-4’), 11.28 (br s, 1H, H-1’).

*3-[2-(2-Amino-4-carboxyphenyl)-1-(phenethylthio)ethyl]-1H-indole-6-carboxylic acid* (**7b**). Prepared according to Procedure A from indole-6-carboxylic acid and 2-phenylethanethiol. Yield 32%; HRMS: M+H^+^ (C_26_H_25_N_2_O_4_S) 461.1548; calculated 461.1535. Elemental analysis: found, %: N 5.26, C 61.88, H 4.74; calculated for 2C_26_H_24_N_2_O_4_S·CF_3_COOH·H_2_O, %: N 5.32, C 61.59, H 4.88.


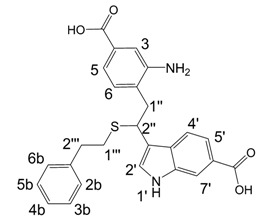


^1^H-NMR (400MHz, DMSO-d_6_, 25°C): δ 2.42 and 2.52-2.63 (2m, 4H, 2H-1’’’, 2H-2’’’), 3.20-3.32 (m, 2H, 2H-1’’), 4.69 (m, 1H, H-2’’), 6.97 (d, *J* = 8.0 Hz, 1H, H-H-6), 7.02 (XX´ part of AA´XX´system, 2H, H-2b, H-6b), 7.02 (m, 1H, H-5), 7.09-7.14 (m, 1H, H-4b), 7.16-7.20 (AA´ part of AA´XX´system, 2H, H-3b, H-5b), 7.29 (d, *J* = 1.6 Hz, 1H, H-3), 7.53 (d, *J* = 2.4 Hz, 1H, H-2’), 7.58 (dd, *J* = 8.4, 1.6 Hz, 1H, H-5’), 7.77 (d, *J* = 8.4 Hz, 1H, H-4’), 7.96 (dd, *J* = 1.6, 0.8 Hz, 1H, H-7’), 11.26 (d, *J* = 2.8 Hz, 1H, H-1’).

*3-{2-[2-Amino-5-(diethylcarbamoyl)phenyl]-1-(phenethylthio)ethyl}-N,N-diethyl-1H-indole-5-carbox-amide* (**7c**). Compound **7c** was prepared according to Procedure A using indole-5-carboxylic acid diethylamide [24] and 2-phenylethanethiol as starting materials. Yield 30%; HRMS: M+H^+^ (C_34_H_43_N_4_O_3_S) 571.3101; calculated 571.3106; Elemental analysis: found, %: N 9.47, C 69.97, H 7.37; calculated for 6C_34_H_42_N_4_O_2_S·CF_3_COOH, %: N 9.50, C 69.92, H 7.21.


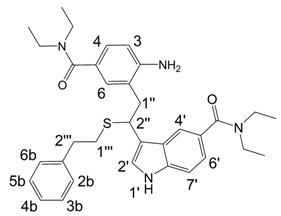


^1^H-NMR (400 MHz, DMSO-d_6_, 25°C): δ 0.87 (m, 6H, 2CH_3_), 1.09 (m, 6H, 2CH_3_), 2.52-2.63 (m, 4H, 2H-1’’’, 2H-2’’’), 3.02 (m, 4H, 2CH_2_ NEt), 3.22 (m, 2H, H-1’’), 3.31 (m, 4H, 2CH_2_ NEt), 4.64(m, 1H, H-2’’), 6.69 (d, *J* = 8.0 Hz, 1H, H-3), 6.77 (d, *J* = 2.0 Hz, 1H, H-6), 6.91 (dd, *J* = 8.0, 2.0 Hz, 1H, H-4), 7.00 (XX´ part of AA´XX´system, 2H, H-2b, H-6b), 7.04 (dd, *J* = 8.4, 1.6 Hz, 1H, H-6’), 7.09-7.13 (m, 1H, 1H-4b), 7.15-7.19 (AA´ part of AA´XX´system, 2H, H-3b, H-5b), 7.33 (d, *J* = 2.4 Hz, 1H, H-2’), 7.34 (d, *J* = 8.4 Hz, 1H, H-7’), 7.71 (m, 1H, 1H-4’), 11.08 (d, *J* = 2.4 Hz, 1H, H-1’).

*3-[2-(2-Amino-5-cyanophenyl)-1-(phenethylthio)ethyl]-1H-indole-5-carbonitrile* (**7d**). **7d** was prepared according to Procedure A from 5-cyanoindole and 2-phenylethanethiol. Yield 15%; HRMS: M+H^+^ (C_26_H_23_N_4_S) 423.1674; calculated 423.1643.


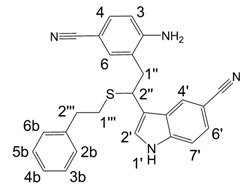


^1^H-NMR (400MHz, DMSO-d_6_, 25°C): δ 2.43 and 2.52-2.64 (2m, 4H, 2H-1’’’, 2H-2’’’), 3.16 (dd, *J* = 8.0, 2.0 Hz, 2H, 2H-1’’), 4.70 (t, *J* = 8.0 Hz, 1H, H-2’’), 6.59 (d, *J* = 8.8 Hz, 1H, H-3), 7.02 (XX´ part of AA´XX´system, 2H, H-2b, H-6b), 7.02 (m, 1H), 7.12-7.23 (AA´ part of AA´XX´system, 2H, H-3b, H-5b), 7.12-7.23 (m, 1H, H-4b), 7.32 (d, *J* = 2.0 Hz, 1H), 7.36-7.44 (m, 1H), 7.47 (m, 1H), 7.54 (d, *J* = 2.4 Hz, 1H), 11.48 (br s, 1H, H-1’).

*4-Fluoro-2-[2-(5-fluoro-1H-indol-3-yl)-2-(phenethylthio)ethyl]aniline* (**7e**). **7e** was prepared according to Procedure A using 5-fluoroindole and 2-phenylethanethiol as starting materials. Yield 2%; HRMS: M+H^+^ (C_24_H_23_F_2_N_2_S) 409.1533; calculated 409.1550.


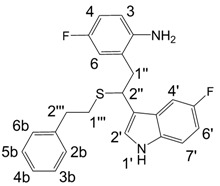


*4-Chloro-2-[2-(5-chloro-1H-indol-3-yl)-2-(phenethylthio)ethyl]aniline* (**7f**). **7f** was prepared according to Procedure A from 5-chloroindole and 2-phenylethanethiol. Yield 5%; HRMS: M+H^+^ (C_24_H_23_Cl_2_N_2_S) 441.0958; calculated 441.0959; Elemental analysis: found, %: N 6.09, C 65.43, H 4.96; calculated for C_24_H_22_Cl_2_N_2_S, %: N 6.35, C 65.30, H 5.02.


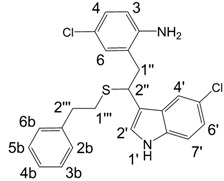


^1^H-NMR (400MHz, DMSO-d_6_, 25°C): δ 2.47 and 2.53 (2m, 2H, 2H-1’’’), 2.62 (m, 2H, 2H-2’’’), 3.14 (m, 2H, 2H-1’’), 4.62 (m, 1H, 1H-2’’), 6.56 (d, *J* = 8.8 Hz, 1H, H-3), 6.83 (dd, *J* = 8.8, 2.8 Hz, 1H, H-4), 6.91 (d, *J* = 2.8 Hz, 1H, H-6), 7.02-7.06 (XX´ part of AA´XX´system, 2H, H-2b, H-6b), 7.02-7.06 (m, 1H, H-6’), 7.12 (m, 1H, H-4b), 7.17-7.22 (AA´ part of AA´XX´system, 2H, H-3b, H-5b), 7.32 (d, *J* = 8.4 Hz, 1H, H-7’), 7.41 (d, *J* = 2.4 Hz, 1H, H-2’), 7.73 (d, *J* = 2.0 Hz, 1H, H-4’), 11.09 (br s, 1H, H-1’).

*3-[2-(2-Aminophenyl)-1-(phenethylthio)ethyl]-1H-indole-5-carboxylic acid* (**9**). Compound **9** was prepared according to Procedure A using indole, indole-5-carboxylic acid (equimolar quantities) and 2-phenylethanethiol as starting materials. Yield 19%; HRMS: M+H^+^ 417.1618. C_25_H_25_N_2_O_2_S; calculated 417.1636. Elemental analysis: found, %: N 5.94, C 64.41, H 5.10; calculated for 2C_25_H_24_N_2_O_2_S· CF_3_COOH·H_2_O, %: N 5.81, C 64.71, H 5.33.


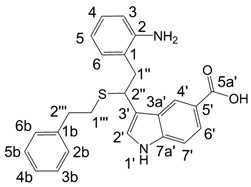


1H-NMR (500 MHz, DMSO-d_6_, 25°C): δ 2.65 (m, 2H, 2H-2’’’), 3.31, 3.35 (m, 2H, 2H-1’’’), 3.31, 3.35 (m, 2H, 2H-1’’), 4.74 (m, 1H, H-2’’), 6.79 (m, 1H, H-3), 6.96(m, 1H, H-5), 7.03 (m, 1H, H-6), 7.05 (m, 1H, H-4), 7.05 (XX´ part of AA´XX´system, 2H, H-2b, H-6b), 7.14 (m, 1H, H-4b), 7.20 (AA´ part of AA´XX´system, 2H, H-3b, H-5b), 7.40 (d, *J* = 8.6 Hz, 1H, H-7’), 7.46 (d, *J* = 2.3 Hz, 1H, H-2’), 7.71 (dd, *J* = 8.6 Hz, 1.5 Hz, 1H, H-6’), 8.46 (s, 1H, H-4’), 11.28 (d, *J* = 1.8 Hz, 1H, H-1’). 13C NMR (125 MHz, DMSO-d_6_, 25°C): δ 31.1 (C-1’’’), 34.9 (C-2’’’), 35.6 (C-1’’), 38.7 (C-2’’), 110.6 (C-7’), 115.2 (C-3’), 118.4 (C-5), 120.2 (C-5’), 121.1 (C-3), 121.4 (C-6’), 121.8 (C-4’), 124.6 (C-2’), 124.7 (C-3a’), 125.2 (C-4b), 126.4 (C-4), 127.4 (C-3b, C-5b), 127.6 (C-2b, C-6b), 129.8 (C-6), 129.9 (C-1), 138.2 (C-7a’), 139.8 (C-1b), 150.0 (C-2), 167.7 (C-5a’).

*3-[2-(2-Aminophenyl)-1-(phenethylthio)ethyl]-2,3-dihydro-1H,1'H-2,3'-biindole-5'-carboxylic acid* (**10**). This compound was prepared according to Procedure A using indole, indole-5-carboxylic acid (equimolar quantities) and 2-phenylethanethiol. Yield 1%; HRMS: M+H^+^ (C_33_H_32_N_3_O_2_S) 534.2212; calculated 534.2215.


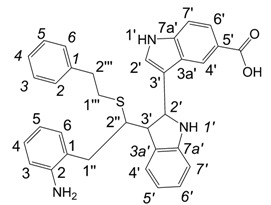


^1^H-NMR (500 MHz, DMSO-d_6_, 25°C): δ 2.23 (m, 2H, 2H-1’’’), 2.30, 2.37 (m, 2H, 2H-2’’’), 2.65 (m, 1H, H_a_-1’’), 2.94 (m, 1H, H_b_-1’’), 3.54 (m, 1H, H-2’’), 3.98 (m, 1H, H-*3’*), 5.24 (d, *J* = 7.6 Hz, 1H, H-*2’*), 6.64 (d, *J* = 7.5 Hz, 1H, H-*7’*), 6.71 (m, 1H, H-*5’*), 6.72 (m, 1H, H-3), 6.78 (XX´ part of AA´XX´system, 2H, H-*2*, H-*6*), 6.91(m, 1H, H-5), 6.93 (m, 1H, H-4), 7.05 (m, 1H, H-6), 7.06 (m, 1H, H-*6’*), 7.11 (AA´ part of AA´XX´system, 2H, H-*3*, H-*5*), 7.14 (m, 1H, H-*4*), 7.31 (d, *J* = 7.3 Hz, 1H, H-*4’*), 7.35 (m, 1H, H-2’), 7.45 (d, *J* = 8.6 Hz, 1H, H-7’), 7.74 (dd, *J* = 8.6 Hz, 1.5 Hz, 1H, H-6’), 8.26 (s, 1H, H-4’), 11.35 (m, 1H, H-1’), 12.36 (br, 1H, COOH); ^13^C-NMR (125 MHz, DMSO-d_6_, 25°C): δ 32.5 (C-1’’’), 34.4 (C-1’’), 34.5 (C-2’’’), 47.1 (C-2’’), 53.1 (C-*3’*), 57.9 (C-*2’*), 108.5 (C-*7’*), 110.6 (C-7’), 117.2 (C-*5’*), 117.5 (C-5), 117.8 (C-3’), 120.3 (C-5’), 121.6 (C-4’), 121.8 (C-6’), 123.7 (C-*4’*), 124.4 (C-2’), 124.5 (C-3a’), 125.2 (C-*4*), 126.9 (C-6), 126.9 (C-*6’*), 127.3 (C-*3, 5*), 127.5 (C-*2*, *6*), 130.5 (C-4), 138.6 (C-7a’), 139.6 (C-*1*), 167.5 (C-COOH).

*3-[2-(2-Aminophenyl)-1-(phenethylthio)ethyl]-1H,1'H-2,3'-biindole-5'-carboxylic acid* (**11**). Compound **11** was synthesized from **10** (5 mg) when the latter was dissolved in DMSO-d_6_ (0.7 mL) and the solution was allowed to stand in a NMR tube at room temperature for one week.


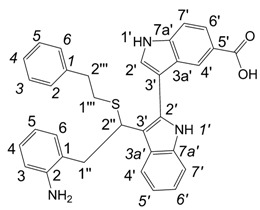


^1^H-NMR (500 MHz, DMSO-d_6_, 25°C): δ 2.21, 2.30 (m, 4H, 2H-1’’’, 2H-2’’’), 3.39 (m, 1H, H_a_-1’’), 3.45 (m, 1H, H_b_-1’’), 4.64 (dd, *J* = 8.9 Hz, 5.9 Hz, 1H, H-2’’), 6.55 (XX´ part of AA´XX´system, 2H, H-*2*, H-*6*), 6.70 (d, *J* = 7.4 Hz, 1H, H-6), 6.80 (m, 1H, H-2’), 6.94 (m, 1H, H-*4*), 6.96 (AA´ part of AA´XX´system, 2H, H-*3*, H-*5*), 7.03 (m, 1H, H-5), 7.03 (m, 1H, H-4), 7.07 (m, 1H, H-*5’*), 7.13 (m, 1H, H-*6’*), 7.42 (d, *J* = 8.0 Hz, 1H, H-*7’*), 7.48 (d, *J* = 8.6 Hz, 1H, H-7’), 7.79 (dd, *J* = 8.6 Hz, 1.5 Hz, 1H, H-6’), 8.02 (d, *J* = 7.9 Hz, 1H, H-*4’*), 8.36 (s, 1H, H-4’), 11.20 (m, 1H, H-*1’*), 11.74 (m, 1H, H-1’), 12.50 (br, 1H, COOH); ^13^C-NMR (125 MHz, DMSO-d_6_, 25°C): δ 31.5 (C-1’’’), 35.1 (C-1’’, 2’’’), 39.9 (C-2’’), 107.9 (C-3’), 109.6 (C-*3’*), 110.7 (C-7’, *7’*), 117.7 (C-*5’*), 119.4 (C-*4’*), 120.2 (C-*6’*), 121.4 (C-5’), 121.9 (C-4’), 122.3(C-6’), 125.0 (C-*4*), 125.2 (C-3a’), 125.3 (C-2’), 125.5 (C-*3a’*), 126.8 (C-4, 5), 127.2 (C-*3, 5*), 127.3 (C-*2*, *6*), 129.6 (C-2), 129.7 (C-6), 130.6 (C-1, *2’*), 136.5 (C-*7a’*), 137.9 (C-7a’), 139.4 (C-*1*), 167.5 (C-COOH).

*Procedure C. Preparation of 3,3'-{1,1'-[ethane-1,2-diylbis(sulfanediyl)]bis[2-(2-acetamido-5-carboxyphenyl)ethane-1,1-diyl]}bis(1H-indole-5-carboxylic acid)* (**6b**). Compound **6a** (6.6 mg, 6.24 µmol) was dissolved in DMF (180 µL) and acetic anhydride (30 µL, 318 µmol) was added. The mixture was allowed to stand for 40 h, diluted with water to 1 mL volume, centrifuged and the clear solution in several portions applied onto an HPLC semipreparative column (10 x 250 mm, Vydac RP C_18_ 90Å Pharmaceutical 201HS1010), eluent - 22 % MeCN in water + 0.1% TFA, flow 5 mL/min, detection at 280 nm. Eluate fractions containing pure putative **6b** were pooled and freeze-dried. Yield ofoff-white powder was 3.4 mg (57%); HRMS: M+H+ (C_42_H_39_N_4_O_10_S_2_) 823.2129; calculated 823.2107. M-H- (C_42_H_37_N_4_O_10_S_2_) 821.1979; calculated 821.1951; Elemental analysis: found, %: N 6.1, C 54.3, H 4.6; calculated for 3C_42_H_38_N_4_O_10_S_2_·2CF_3_COOH·9H_2_O, %: N 5.88. C 54.62, H 4.72.


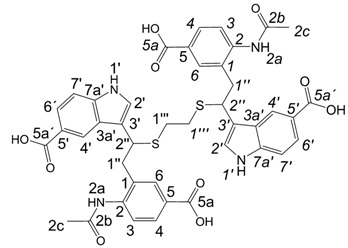


^1^H-NMR (500 MHz, DMSO-d_6_, 25°C): δ 1.88, 1.90 (2s, 6H, H-2c, H-*2c*), 2.3 – 2.5 (m, 4H, 2H-1’’’, 2H-*1’’’*), 3.25 - 3.40 (m, 4H, 2H-1’’, 2H-*1’’*), 4.42, 4.44 (2m, 2H, H-2’’, H-*2’’*), 7.19 (m, 2H, H-2’, H-*2’*), 7.33 (dd, *J* = 8.5 Hz, 2.7 Hz, 2H, H-7’, H-*7’*), 7.45 (d, *J* = 8.6 Hz, 2H, H-3, H-*3*), 7.64 (m, 2H, H-4, H-*4*), 7.66 (m, 2H, H-6, H-*6*), 7.68 (m, 2H, H-6’, H-*6’*), 8.38, 8.39 (2m, 2H, H-4’, H-*4’*), 9.35, 9.36 (2s, 2H, H-2a, H-*2a*), 11.14 (m, 2H, H-1’, H-*1’*), 12.5 (br, 2H, H-5a, H-*5a*); ^13^C NMR (125 MHz, DMSO-d_6_, 25°C): δ 22.4(C-2c, C-*2c*), 30.1 (C-1’’’, C-*1’’’*), 36.3 (C-1’’, C-*1’’*), 40.5 (C-2’’, C-*2’’*), 110.6 (C-7’, C-*7’*), 114.5 (C-3’, C-*3’*), 120.4 (C-5’, C-*5’*), 121.6 (C-4’, C-*4’*), 126.1 (C-5, C-*5*), 121.8 (C-6’, C-*6’*), 124.3 (C-3a’, C-*3a’*), 124.6 (C-2’, C-*2’*), 126.9 (C-4, C-*4*), 131.0 (C-6, C-*6*), 132.3 (C-1, C-*1*), 138.6 (C-7a’, C-*7a’*), 140.0 (C-2, C-*2*), 166.2 (C-5a, C-*5a*), 167.8 (C-5a’, C-*5a’*), 167.8 (C-2b, C-*2b*).

The following compounds were similarly prepared by Procedure C:

*1-Acetyl-2,3-dihydro-1H,1'H-2,3'-biindole-5,5'-dicarboxylic acid* (**2b**). From **2a**. HRMS: M+H^+^ (C_20_H_17_N_2_O_5_) 365.1138; calculated 365.1137; M-H^-^ (C_20_H_15_N_2_O_5_) 363.0961; calculated 363.0981; Elemental analysis: found, %: N 6.3, C 56.1, H 4.2; calculated for 2C_20_H_16_N_2_O_5_·CF_3_COOH·3H_2_O, %: N 6.25. C 56.25, H 4.38.


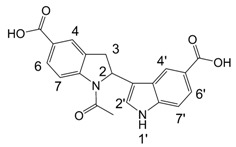


^1^H-NMR (400 MHz, DMSO-d_6_, 25°C): δ 2.07 (s, 3H, CH_3_), 3.01 (d, *J* = 16.0 Hz, 1H, H-3), 3.79 (dd, *J* = 16.0, 10.4 Hz, 1H, H-3), 6.04 (dd, *J* = 10.0, 2.0 Hz, 1H, H-2), 7.17 (d, *J* = 2.0 Hz, 1H, H-2’), 7.40 (d, *J* = 8.8 Hz, 1H, H-7), 7.69 (dd, *J* = 8.8, 1.6 Hz, 1H, H-6), 7.77 (d, *J* = 1.6 Hz, 1H, H-4), 7.88 (dd, *J* = 8.4, 2.0 Hz, 1H, H-6’), 7.98 (s, 1H, H-4’), 8.18 (m, 1H, H-7’), 11.34 (br s, 1H, H-1’).

*3,3'-[2-(2-Acetamido-5-carboxyphenyl)ethane-1,1-diyl]bis(1H-indole-5-carboxylic acid)* (**3b**). From **3a**. HRMS: M+H^+^ (C_29_H_24_N_3_O_7_) 526.162, calculated 526.1614. M-H^-^ (C_29_H_22_N_3_O_7_) 524.1467, calculated 524.1458; Elemental analysis: found, %: N 6.2, C 53.4, H 4.5; calculated for C_29_H_23_N_3_O_4_·CF_3_COOH·3H_2_O, %: N 6.06. C 53.68, H 4.36.


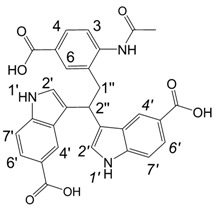


^1^H-NMR (400 MHz, DMSO-d_6_, 25°C): δ 1.88 (s, 3H, CH_3_), 3.54 (d, *J* = 7.6 Hz, 2H, 2H-1’’), 4.83 (dd, *J* = 7.6, 7.6 Hz, 1H, 1H-2’’), 7.27 (d, *J* = 2.4 Hz, 2H, H-1’, H-*1’*), 7.31 (d, *J* = 8.8 Hz, 2H, H-7’, H-*7’*), 7.48 (d, *J* = 8.4 Hz, 1H, H-3), 7.59 (dd, *J* = 8.8, 1.6 Hz, 2H, H-6’, H-*6’*), 7.61 (dd, *J* = 8.4, 2.0 Hz, 1H, H-4), 7.69 (d, *J* = 2.0 Hz, 1H, H-6), 8.11 (d, *J* = 1.6 Hz, 2H, H-4’, *H-4’*), 9.31 (s, 1H, H-2a), 11.13 (d, *J* = 2.4 Hz, 2H, H-1’, *H-1’*).

*3-[2-(2-Acetamido-5-carboxyphenyl)-1-(2-mercaptoethylthio)ethyl]-1H-indole-5-carboxylic acid* (**5b**). From **5a**. HRMS: M+H^+^ (C_22_H_23_N_2_O_5_S_2_) 459.1037; calculated 459.1048; Elemental analysis: found, %: N 5.70, C 56.39, H 4.81; calculated for 8C_22_H_22_N_2_O_5_S_2_·CF_3_COOH, %: N 5.92. C 56.52, H 4.72.


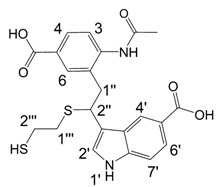


^1^H-NMR (400 MHz, DMSO-d_6_, 25°C): δ 1.95 (s, 3H, CH_3_), 2.34(m, 1H, SH), 2.46 and 2.53 (2m, 4H, 2H-1’’’ and 2H-2’’’), 3.34 (m, 1H, 1H-1’’), 3.42 (dd, *J* = 14.4, 8.4 Hz, 1H, 1H-1’’), 4.49 (m, 1H, 1H-2’’), 7.31 (d, *J* = 2.4 Hz, 1H, H-2’), 7.36 (d, *J* = 8.8 Hz, 1H, H-7’), 7.45 (d, *J* = 8.4 Hz, 1H, H-3), 7.67 (m, 1H, H-4), 7.69 (dd, *J* = 8.4, 1.6 Hz, 1H, H-6’), 7.73 (d, *J* = 1.6 Hz, 1H, H-6), 8.41 (d, *J* = 1.6 Hz, 1H, H-4’), 9.44 (s, 1H, H-2a), 11.23 (d, *J* = 2.0 Hz, 1H, H-1’).

*3-[2-(2-Acetamido-5-carboxyphenyl)-1-(phenethylthio)ethyl]-1H-indole-5-carboxylic acid* (**8a**). From **7a**. HRMS: M+H^+^ (C_28_H_27_N_2_O_5_S) 503.1636; calculated 503.1640; Elemental analysis: found, %: N 5.17, C 66.28, H 5.44; calculated for 3C_28_H_26_N_2_O_5_S·H_2_O, %: N 5.51. C 66.12, H 5.28.


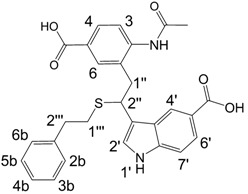


^1^H-NMR (270 MHz, DMSO-d_6_, 25°C): δ 1.92 (s, 3H, CH_3_), 2.55 and 2.60 (2m, 4H, 2H-1’’’, 2H-2’’’), 3.30-3.49 (m, 2H, 2H-1’’), 4.48 (m, 1H, H-2’’), 7.03 (XX´ part of AA´XX´system, 2H, H-2b, H-6b), 7.08-7.22 (m, 1H, H-4b), 7.08-7.22 (AA´ part of AA´XX´system, 2H, H-3b, H-5b), 7.32 (d, *J* = 2.3 Hz, 1H, H-2’), 7.37 (d, *J* = 8.6 Hz, 1H, H-7’), 7.47 (d, *J* = 8.3 Hz, 1H, H-3), 7.69 (m, 2H, H-4, H-6’), 7.75 (d, *J* = 1.3 Hz, 1H, H-6), 8.43 (d, *J* = 1.3 Hz, 1H, H-4’), 9.46 (br s, 1H, H-2a), 11.25 (d, *J* = 2.3 Hz, 1H, H-1’).

*3-[2-(2-Acetamido-4-carboxyphenyl)-1-(phenethylthio)ethyl]-1H-indole-6-carboxylic acid* (**8b**). From **7b**. HRMS: M+H^+^ (C_28_H_27_N_2_O_5_S) 503.1656; calculated 503.1640; Elemental analysis: found, %: N 5.30, C 65.24, H 5.17; calculated for 4C_28_H_26_N_2_O_5_S·3H_2_O, %: N 5.43. C 65.16, H 5.37.


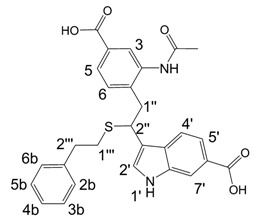


^1^H-NMR (270 MHz, DMSO-d_6_, 25°C): δ 1.97 (s, 3H, CH_3_), 2.54 and 2.61 (2m, 4H, 2H-1’’’, 2H-2’’’), 3.31-3.51 (m, 2H, 2H-1’’), 4.50 (m, 1H, H-2’’), 7.04 (XX´ part of AA´XX´system, 2H, H-2b, H-6b), 7.09-7.24 (m, 1H, 4b), 7.09-7.24 (AA´ part of AA´XX´system, 2H, H-3b, H-5b), 7.09-7.24 (m, 1H, H-6), 7.42 (d, *J* = 2.6Hz, 1H, H-2’), 7.51 (dd, *J* = 7.9, 2.0 Hz, 1H, H-5), 7.57(dd, *J* = 8.3, 1.6 Hz, 1H, H-5’), 7.73 (d, *J* = 8.3 Hz, 1H, H-4’), 7.84 (d, *J* = 2.0 Hz, 1H, H-3), 7.96 (d, *J* = 1.6 Hz, 1H, H-7’), 9.48 (br s, 1H, H-2a), 11.27 (d, *J* = 2.3 Hz, 1H, H-1’).

*3-{2-[2-Acetamido-5-(diethylcarbamoyl)phenyl]-1-(phenethylthio)ethyl}-N,N-diethyl-1H-indole-5-carboxamide* (**8c**). From **7c**. HRMS: M+H^+^ (C_36_H_45_N_5_O_3_S) 613.3205; calculated 613.3212; Elemental analysis: found, %: N 8.63, C 69.01, H 7.03; calculated for 6C_36_H_44_N_5_O_3_S·CF_3_COOH H_2_O, %: N 8.83. C 68.74, H 7.07.


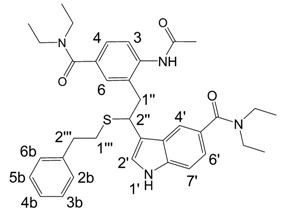


^1^H-NMR (400 MHz, DMSO-d_6_, 25°C): δ 0.74 and 1.08 (2m, 12H, 4CH3), 1.99 (s, 3H, COCH_3_), 2.51-2.66 (m, 4H, 2H-1’’’, 2H-2’’’), 3.24-3.44 (m, 10H, 4CH_2_ NEt, 2H-1’’), 4.46 (dd, *J* = 8.8, 6.4 Hz, 1H, H-2’’), 6.89 (d, *J* = 2.0 Hz, 1H, H-6), 7.02-7.06 (XX´ part of AA´XX´system, 2H, H-2b, H-6b), 7.02-7.06 (m, 2H, H-4, H-6’), 7.09-7.13 (m, 1H, H-4b), 7.16-7.20 (AA´ part of AA´XX´system, 2H, H-3b, H-5b), 7.23 (d, *J* = 2.4 Hz, 1H, H-2’), 7.31 (d, *J* = 8.0 Hz, 1H, H-3), 7.32 (d, *J* = 8.4 Hz, 1H, H-7’), 7.68 (m, 1H, H-4’), 9.45 (br s, 1H, H-2a), 11.06 (d, *J* = 2.4 Hz, 1H, H-1’).
